# The efficacy and safety of nirsevimab for the prevention of RSV infection among infants: A systematic review and meta-analysis

**DOI:** 10.3389/fped.2023.1132740

**Published:** 2023-04-04

**Authors:** Maria Wilma R. Turalde-Mapili, Jerahmeel Aleson L. Mapili, Christian Wilson R. Turalde, Marimel R. Pagcatipunan

**Affiliations:** ^1^ Department of Pediatrics, College of Medicine, Philippine General Hospital, University of the Philippines Manila, Manila, Philippines; ^2^ Department of Medicine, College of Medicine, Philippine General Hospital, University of the Philippines Manila, Manila, Philippines; ^3^ Department of Physiology, College of Medicine, Philippine General Hospital, University of the Philippines Manila, Manila, Philippines; ^4^ National Teacher Training Center for the Health Professions, University of the Philippines Manila, Manila, Philippines

**Keywords:** respiratory syncytial virus, nirsevimab, lower respiratory tract infection, infants, prevention

## Abstract

**Background:**

Respiratory syncytial virus (RSV) is a leading cause of morbidity and mortality among infants with a global incidence of 9.5% and a mortality rate of 2.2%. The management of RSV infection is mainly supportive and, aside from emerging monoclonal antibodies, there has been no benefit of most preventive measures. Recent evidence suggests the potential of nirsevimab in preventing RSV infection.

**Objective:**

This study aims to determine the efficacy and safety of nirsevimab in preventing RSV infection among infants using a review of relevant clinical trials.

**Methods:**

We performed a random-effects meta-analysis among infants comparing nirsevimab injection vs. placebo. MEDLINE, CENTRAL, Scopus, and ClinicalTrials.gov were searched for relevant trials from inception to June 2022. The selected studies were assessed for risk of bias using the Revised Cochrane Risk-of-Bias (RoB2) tool and for quality of evidence using the Grades of Recommendation, Assessment, Development and Evaluation (GRADE) approach.

**Results:**

Two studies were included. Data analysis showed that among infants, nirsevimab given before the RSV season significantly reduced the risk of medically attended RSV-related infection (RR: 0.26; 95% CI: 0.18–0.38) and the risk of hospitalization due to RSV infection (RR: 0.24; 95% CI: 0.13–0.47). There was no difference in terms of adverse events leading to death (RR: 0.78, 95% CI: 0.20–2.98) and adverse events of special interest (RR: 0.92, 95% CI: 0.25–3.38).

**Conclusions:**

The use of nirsevimab to prevent RSV infections and hospitalization shows its promising potential, but studies on its cost-effectiveness are lacking. We recommend that further studies be done to look into the applicability and cost-effectiveness of nirsevimab.

## 1. Introduction

Respiratory syncytial virus (RSV) is a leading cause of morbidity among infants and young children. The greatest burden of severe disease requiring hospitalization is among infants under one year (1). The estimated global incidence rate of RSV-associated acute lower respiratory tract infections (LRTI) among infants in a recent systematic review is 94.6 per 1,000 infants per year leading to 15.9 admissions per 1,000 infants per year and a mortality rate of 2.2% (2).

The treatment of RSV infection remains mainly supportive in most clinical settings with oxygen or ventilatory support and nutritional upbuilding serving as the cornerstone of management. Evidence regarding the medical management of RSV infection is bleak as there have been no proven clinical benefit regarding the use of bronchodilators, corticosteroids, antibiotics, nebulized epinephrine, leukotriene inhibitors, nebulized hypertonic saline or chest physiotherapy to reduce morbidity or mortality (3). Ribavirin has also been advocated for its use in bronchiolitis, but recent evidence suggests that it has no clinical benefit in bronchiolitis caused specifically by RSV (4). Intravenous immunoglobulin (IVIG) has also been explored for the treatment of RSV infection but has likewise failed to show significant clinical benefit (5). Consequently, there is currently no recommendation regarding which drugs can be used for the treatment of RSV associated LRTI.

Despite the lack of evidence regarding its treatment, there is growing evidence regarding drugs which can be used for its prevention. Currently there are no available maternal or infant RSV vaccines. However, there is a growing body of evidence regarding the efficacy and safety of monoclonal antibodies for the prevention of RSV infection among infants and young children. In one Cochrane review, palivizumab resulted in a significant reduction in overall hospitalizations and respiratory disease-related hospitalizations but failed to show mortality benefit when compared to placebo among patients given the drug before the RSV season (6). Due to the evidence for reducing hospitalizations, palivizumab has been recommended in the United States of America and the United Kingdom as the only drug which can be used for the prevention of RSV. In another trial by the Motavizumab Study Group, motavizumab, another monoclonal antibody like palivizumab, has also been shown to be non-inferior to palivizumab for the prevention of RSV among high-risk infants (7). However, motavizumab was associated with an increase in cutaneous hypersensitivity reactions in recipients and this led to the Food and Drug Administration (FDA) not granting licensure.

Despite the strong evidence regarding the use of these two monoclonal antibodies as immunoprophylactic agents, there have been issues regarding its high cost, selling at around £5,000 for its full course, and consequently raises questions regarding its cost-effectiveness. A systematic review has suggested that palivizumab's cost-effectiveness is restricted only at a willingness-to-pay threshold of £30,000 per quality-adjusted life year (8). Aside from its cost, there have also been questions regarding the adherence to the full regimen as these drugs have been dosed to be given at monthly intervals for five months. Hence, it is imperative to investigate an accessible and cost-effective agent with promising potential for preventing RSV infection among infants.

Nirsevimab, a recombinant human monoclonal antibody that binds to the F1 and F2 subunits of the F protein of the RSV to prevent viral cellular invasion, is an emerging alternative preventive agent against RSV infection. A recent study involving animal models shows that nirsevimab has higher potency and longer half-life compared to motavizumab and palivizumab (9).

Recently, there have been randomized clinical trials regarding the efficacy and safety of nirsevimab, a relatively newer monoclonal antibody targeted against RSV. Nirsevimab, administered as a single intramuscular dose, provides a promising alternative to the previously recommended palivizumab or motavizumab.

This study aims to determine the efficacy and safety of nirsevimab in preventing RSV infection among infants using a review of relevant clinical trials. Specifically, this study aims to determine the

1. Efficacy of nirsevimab in terms of preventing incidence of the following: RSV illness diagnosis, RSV-related hospitalizations, and any LRTI among infants; and
2. Safety of nirsevimab in terms of life threatening or fatal adverse events graded using the National Cancer Institute Common Terminology and Criteria for Adverse Events, and adverse events of special interest.

## 2. Methods

This systematic review and meta-analysis adhere to the PRISMA (Preferred Reporting Items for Systematic Reviews and Meta-Analyses) consensus guidelines (10).

### 2.1. Criteria for selection of studies

Studies with the following criteria were included in this systematic review and meta-analysis: (a) study design is a randomized controlled trial, (b) intervention is only nirsevimab, (c) population includes infants aged one year old and below, (d) efficacy outcomes reported should include RSV infection, RSV-related hospitalization, and any respiratory illness, (e) safety outcomes reported should include death, life-threatening adverse events, and adverse events of special interest, and (f) studies should be written in English or with an available English translation. Studies published from the dates of inception of the databases to June 2022 were included in this review. Studies were excluded if: (a) study design is not a randomized controlled trial, (b) comparison is not placebo, (c) outcomes are others not specified in the inclusion criteria.

### 2.2. Outcome measures considered

The primary efficacy outcome is the incidence of RSV illness and the secondary efficacy outcomes are incidence of RSV-related hospitalizations and any respiratory illness. The primary safety outcome is the incidence of life-threatening or fatal adverse events and the secondary safety outcomes are the adverse events of special interest (hypersensitivity, immune complex disease, or thrombocytopenia).

### 2.3. Search methods for the identification and selection of studies

The following databases were searched: MEDLINE by PubMed, Cochrane Central Register for Controlled Trials (CENTRAL), Scopus, and ClinicalTrials.gov website. The following general and MeSH term-based search strategy was employed: (nirsevimab OR “MED18897” [MeSH] OR “MED-18897” [MeSH]) AND (“Respiratory Syncytial Virus Infection” [MeSH] OR “Infections, Respiratory Syncytial Virus” [MeSH] OR “Human respiratory syncytial virus” [MeSH]) AND prevention AND “infants, newborn” [MeSH].

After the initial electronic search, the two investigators (MT and JM) independently reviewed for duplicates to complete the identification phase. The abstracts and other details were reviewed during the screening phase. Studies which do not meet the criteria and objectives of this review were excluded during the screening phase. Studies which meet the inclusion criteria but are identified to have at least one exclusion criterion were still included in the eligibility phase and were subjected to full-text review. After screening for titles and abstracts, the remaining studies underwent full-text review. Studies which meet the inclusion and do not meet the exclusion criteria were included in the final meta-analysis. The discrepancies between the independent literature searches were resolved by discussion with the third and fourth investigators (CT and MP).

### 2.4. Assessment of risk of bias, data collection, and analysis

The risk of bias in each study was independently assessed by two investigators (MT and JM) using the Revised Cochrane Risk-of-Bias Tool for Randomized Trials (RoB2) and were tabulated accordingly (11). The risk of bias table is color-coded as follows: green for low risk, orange for unknown risk, and red for high risk; an explanation for the risk of bias is provided in the tabulation.

Two investigators (MT and JM) independently reviewed the included article for data extraction of all study variables as laid out below. The discrepancies between the independent review were resolved by discussion with the third and fourth investigators to reach a consensus. The following details were extracted from each included study and tabulated: study design, characteristics of the study population, intervention and control in their specified dosing schedules and routes of administration, and the number of events pertaining to each of the selected efficacy and safety outcomes. The data were extracted from the full text articles including their appendices and supplementary files (if applicable).

Overall confidence in the estimates for each outcome were independently assessed by two investigators (MT and JM) based on the Grades of Recommendation, Assessment, Development and Evaluation (GRADE) Working Group system for limitations in study design, evidence directness, consistency, precision of results and publication bias (12).

All statistical analyses were conducted using Review Manager version 5.4 from Cochrane. Relative Risk (RR) was used as a summary statistic for dichotomous outcomes. The results of each study were presented in the Forest plots with summary statistics, 95% confidence intervals and relative weights represented by the middle of the square, the horizontal line, and the relative size of the square, respectively.

For the overall summary statistic, the composite relative risk and 95% confidence interval were represented by the middle and width of the diamond, respectively. The *I*^2^ statistic was used to estimate heterogeneity across studies, with values at 40%–70% with moderate heterogeneity and greater than 70% considered as substantial heterogeneity. In this meta-analysis, the investigators used a random-effects model to take into account the methodological variation across studies.

## 3. Results

### 3.1. Included studies

**Figure 1:**
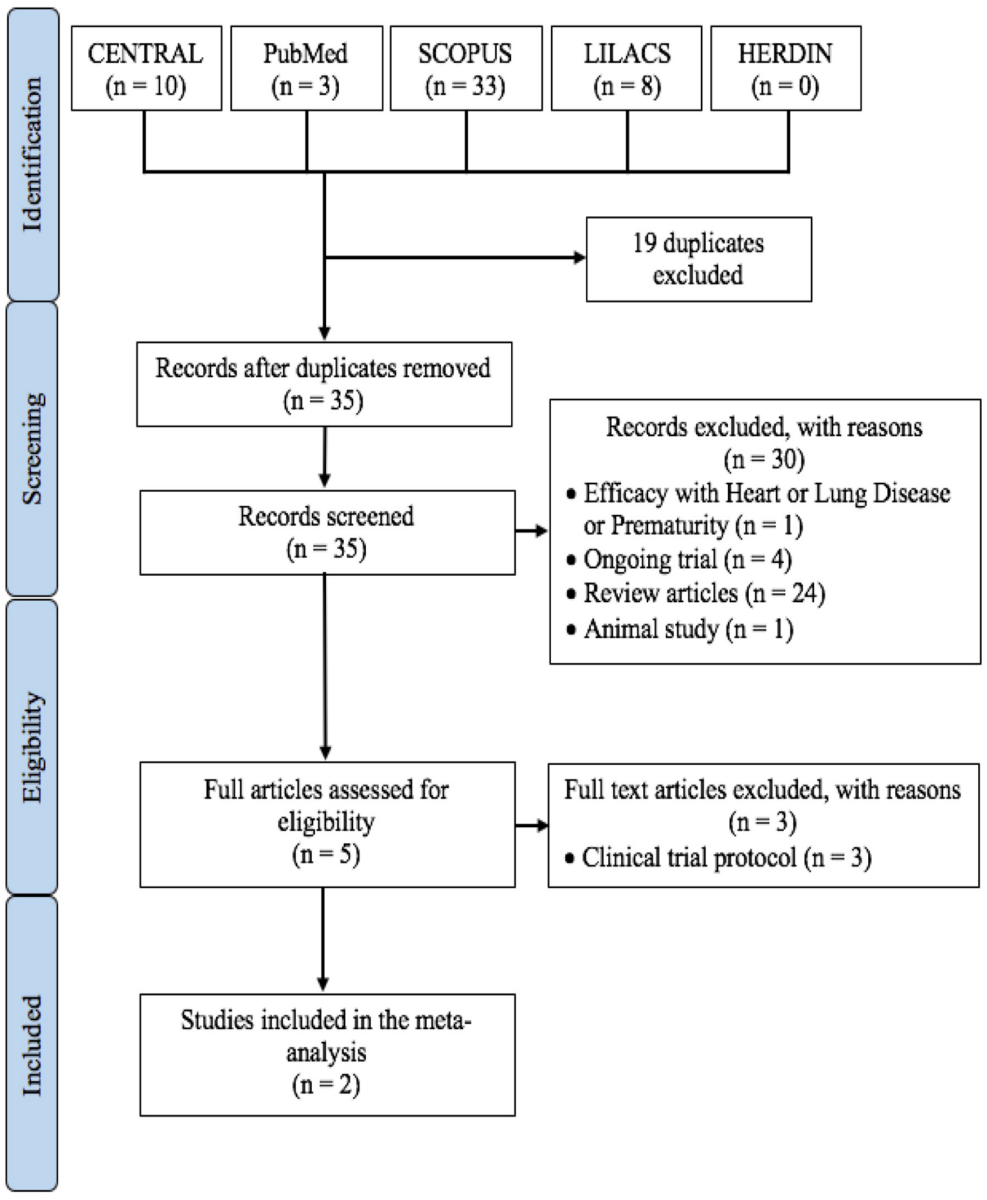
PRISMA summary of literature search.

The final selection of studies for inclusion in this meta-analysis is summarized in Figure 1. The two studies were both multicenter with the Nirsevimab Study Group study in 2020 (13) across 23 countries in Europe, United States, and South Africa and the MELODY Study in 2022 (14) across 20 countries in Europe, United States, South Africa, and Asia.

### 3.2. Population characteristics in the included studies

This meta-analysis included a total population of 2,943 infants, 1,963 from the treatment arm and 980 from the placebo arm. The baseline characteristics were similar between each of the included studies and in the overall comparison (refer to Table 1).

**Table 1:** Baseline characteristics.

	Age	Female sex (*N*, %)	Weight (kg)
**Nirsevimab Study (*N* = 1,453)**			
Intervention *N* = 969	≤3 months 516 (53.3)	468 (48.3)	4.60 ± 1.92
	>3 to ≤6 months 320 (33.0)		
	>6 months 133 (13.7)		
Control *N* = 484	≤3 months 257 (53.1)	224 (46.3)	4.51 ± 1.96
	>3 to ≤6 months 153 (31.6)		
	>6 months 74 (15.3)		
**MELODY Trial (*N* = 1,490)**			
Intervention *N* = 994	≤3.0 months 577 (58.0)	464 (46.8)	<5 kg 403 (40.6)
	>3.0 to ≤6.0 months 317 (31.9)		≥5 kg 589 (59.4)
	>6.0 months 100 (10.1)		
Control *N* = 496	≤3.0 months 285 (57.5)	257 (51.8)	<5 kg 192 (38.7)
	>3.0 to ≤6.0 months 162 (32.7)		≥5 kg 304 (61.3)
	>6.0 months 49 (9.9)		
**Overall (*N* = 2,943)**			
Intervention *N* = 1963	≤3 months 1,093 (55.68)	721 (47.47)	N/A
	>3 to ≤6 months 637 (32.45)		
	>6 months 233 (11.87)		
Control *N* = 980	≤3 months 542 (55.31)	481 (49.08)	N/A
	>3 to ≤6 months 320 (32.14)		
	>6 months 123 (12.55)		

### 3.3. Characteristics of individual studies

A summary of the characteristics of the individual studies included in this meta-analysis are summarized in Table 2.

**Table 2:** Characteristics of individual studies.

Trial	Study Design	Inclusion Criteria	Exclusion Criteria	Intervention	Control	Efficacy Outcomes	Safety Outcomes
Nirsevimab Study (2020)	Randomized control trial	Participants were healthy infants who had been born preterm (gestational age of 29 weeks 0 days through 34 weeks 6 days) and who were 1 year of age or younger and entering their first full RSV season	Participants were excluded if they met local, national, or AAP recommended guidelines to receive RSV prophylaxis, had an acute illness at the time of randomization, had previously had an RSV infection, or had received palivizumab or any other investigational RSV monoclonal antibody or vaccine, including maternal vaccines	One intramuscular injection of 50 mg of nirsevimab	Normal saline placebo	Medically-attended RSV infection RSV-associated hospitalization	Fatal adverse events Special adverse events such as hypersensitivity, immune complex disease, and thrombocytopenia
MELODY (2022)	Randomized control trial	Healthy infants who had been born at a gestational age of at least 35 weeks 0 days, were 1 year of age or younger, and were entering their first RSV season	Participants were excluded if they met national or local criteria to receive commercial palivizumab, had any fever or acute illness within 7 days before randomization, or had RSV infection before or at the time of randomization	One intramuscular injection of nirsevimab with the following dosages: • 50 mg for <5 kg • 100 mg for ≥5 kg	Normal saline placebo	Medically-attended RSV infection RSV-associated hospitalization	Fatal adverse events Special adverse events such as hypersensitivity, immune complex disease, and thrombocytopenia

### 3.4. Risk of bias evaluation

This meta-analysis was analyzed for risk of bias using the Cochrane RoB2 Tool by two investigators as mentioned. A summary of the findings in the risk of bias analysis is tabulated in Table 3. Based on the RoB2 Tool, there was low risk of bias in terms of selection, performance, detection, attrition, and reporting bias. Overall, the studies had a low risk of bias.

**Table 3:** Risk of bias using RoB2 tool.

Author/Trial (Year)	Selection bias				Performance bias		Detection bias		Attrition bias		Reporting bias		Other biases	
	Random sequence generation		Allocation concealment		Blinding of Participants		Blinding of outcome assessment		Loss to follow-up		Selective outcome reporting?		Funding?	
Nirsevimab Study (2020)	Low	Central randomization	Low	Central randomization	Low	Placebo-trial	Low	Blinded	Low	<10%	Low	trial registered	Low	Not for-profit
MELODY (2022)	Low	Central randomization	Low	Central randomization	Low	Placebo-trial	Low	Blinded	Low	<10%	Low	trial registered	Low	Not for-profit

### 3.5. Effects of intervention

#### 3.5.1. Medically-attended RSV-associated LRTI

**Figure 2:**
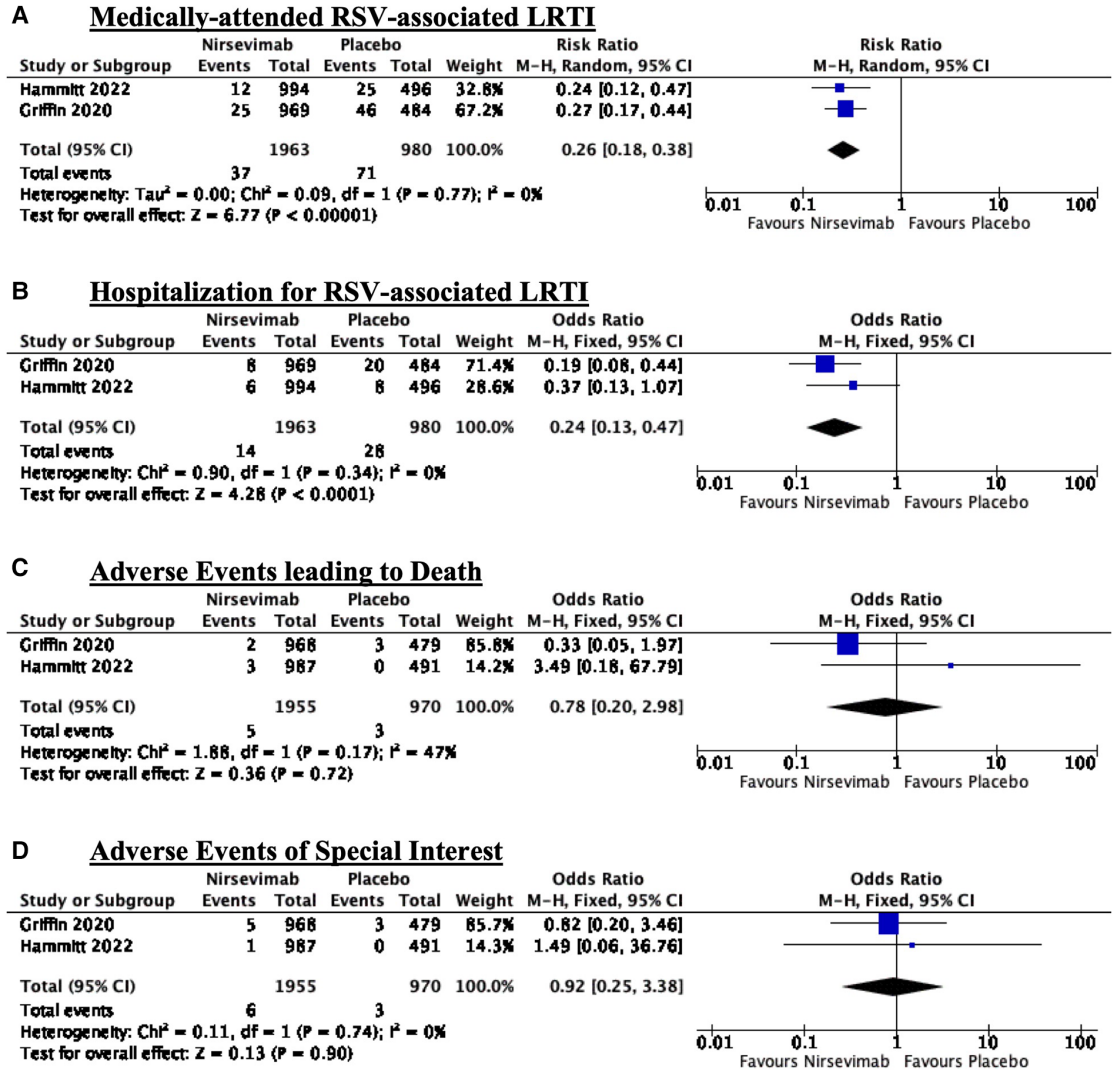
Forest plots summarizing the effects of intervention: (A) Medically-attended RSV-associated LRTI; (B) Hospitalization for RSV-associated LRTI; (C) Adverse events leading to death; and (D) Adverse events of special interest.

Nirsevimab given before the RSV season significantly reduced the risk of medically-attended RSV-associated LRTI (RR: 0.26; 95% CI: 0.18–0.38). There was no significant heterogeneity with an *I*^2^ = 0% (refer to Figure 2A).

#### 3.5.2. Hospitalization for RSV-associated LRTI

Nirsevimab given before the RSV season significantly reduced the risk of hospitalization for RSV-associated LRTI (RR: 0.24; 95% CI: 0.13–0.47). There was no significant heterogeneity with an *I*^2^ = 0% (refer to Figure 2B).

#### 3.5.3. Adverse events leading to death

There is no significant difference between adverse events leading to death between the nirsevimab and placebo arms (RR: 0.78, 95% CI: 0.20–2.98). There was moderate heterogeneity with an *I*^2^ = 47% (refer to Figure 2C).

#### 3.5.4. Adverse events of special interest

There is no significant difference between adverse events of special interest between the nirsevimab and placebo arms (RR: 0.92, 95% CI: 0.25–3.38). There was no significant heterogeneity with an *I*^2^ = 0% (refer to Figure 2D).

### 3.6. Quality of evidence evaluation

Based on the researchers’ assessment of the evidence using the GRADE approach, all outcomes except Adverse Events leading to Death had high quality of evidence. The Adverse Events leading to Death outcome had moderate quality of evidence mainly attributed to inconsistency and moderate heterogeneity. A summary of findings table is seen in Table 4.

**Table 4:** Summary of findings and GRADE quality of evidence.

Outcomes	No of participants (studies) Follow-up	Certainty of the evidence (GRADE)	Relative effect (95% CI)	Anticipated absolute effects	
				Risk with Placebo	Risk difference with Nirsevimab
Medically-attended RSV-associated LRTI (RSV LRTI)	2,943 (2 RCTs)	⊕⊕⊕⊕ High	**RR 0.26** (0.18 to 0.38)	72 per 1,000	**54 fewer per 1,000** (59 fewer to 45 fewer)
Hospitalization for RSV-associated LRTI (RSV Hospitalization)	2,943 (2 RCTs)	⊕⊕⊕⊕ High	**RR 0.24** (0.13 to 0.47)	29 per 1,000	**22 fewer per 1,000** (25 fewer to 15 fewer)
Adverse Events leading to Death (Adverse Event Death)	2,925 (2 RCTs)	⊕⊕⊕○ Moderate	**RR 0.78** (0.20 to 2.98)	3 per 1,000	**1 fewer per 1,000** (2 fewer to 6 more)
Adverse Events of Special Interest	2,925 (2 RCTs)	⊕⊕⊕⊕ High	**RR 0.92** (0.25 to 3.38)	3 per 1,000	**0 fewer per 1,000** (2 fewer to 7 more)

## 4. Discussion

This review provides comprehensive evidence on the efficacy of nirsevimab in preventing RSV-associated LRTI and in decreasing hospitalization for RSV-associated LRTI from pooled results of two studies. In terms of safety, there is no significant increase in risk of adverse events leading to death or adverse events of special interest. This meta-analysis has shown a 74% reduction in the incidence of medically treated RSV-associated LRTI. It should be noted that the markedly glaring significance in reduction in hospitalization at 76% was mostly attributed to the Nirsevimab Study Group trial in 2020 (13) which showed an 81% reduction in RSV-related hospitalizations. Furthermore, it can be noted that the MELODY Trial in 2022 (14) showed no significant reduction in RSV-associated hospitalizations albeit with a trend towards benefit (RR: 0.37, 95% CI: 0.13–1.07). As shown in the comparison of the populations of the two studies in this meta-analysis, it should be noted that the patients enrolled in the Nirsevimab Study Group trial were all preterm infants while the MELODY trial enrolled both late preterm and term infants. It is important to note that there is an ongoing clinical trial (National Clinical Trial number NCT05437510) investigating the efficacy and safety of single intramuscular dose of nirsevimab for the prevention of RSV-associated hospitalizations among healthy term and preterm infants.

The meta-analysis shows that the risk of death and risk of special events of nirsevimab are not statistically significant between the two treatment groups. However, it should be noted that both trials showed differences in the trends of the correlation in this specific outcome with the Nirsevimab Study Group trial showing a trend towards increased risk for nirsevimab. Furthermore, it should be noted that there is moderate heterogeneity (*I*^2^ = 47%) in terms of the outcomes on adverse events leading to death. These nuances may be explained mainly due to the differences in population of each study as already highlighted above. As previously mentioned, a certain subgroup of the total population in this meta-analysis were preterm infants wherein organ prematurity and rapid development changes affect pharmacokinetics and pharmacodynamics which put neonates at an increased risk of adverse drug reactions compared to term infants (15).

In summary, this meta-analysis provides evidence to conclude that nirsevimab reduces the risk of RSV-related disease and hospitalizations across the whole spectrum of infancy regardless of term status at birth. However, the subtle nuances in the benefit between the two trials begs the question if only preterm infants will benefit from nirsevimab injection. On the corollary, preterm infants are also the ones who may potentially be more at risk of adverse events, but this meta-analysis shows no significant difference between the two groups.

Among infants who were both term and preterm, nirsevimab injection before the RSV season significantly reduces the rates of infection and hospitalization related to RSV. There is no significant effect in terms of adverse events leading to death and special adverse events. Indeed, the use of nirsevimab to prevent RSV infections and hospitalization shows its promising potential but studies on its cost-effectiveness are lacking. In a study by Kieffer and colleagues in 2022, it was estimated using a static model that universal immunization of all infants with nirsevimab is expected to reduce 290,174 RSV-MALRTI, 24,986 hospitalizations, and expenditures of $612 million USD (16). We therefore recommend that further studies be done to look into the applicability and cost-effectiveness of nirsevimab.

The results of this meta-analysis also have implications on basic and graduate medical education. Treatment recommendations are continuously being updated and revised as pooled evidence from relevant studies are established. A recent analytic survey emphasizes that medical textbooks may contain outdated treatment recommendations as there is significant lag time from publication of evidence to incorporation of such evidence into medical textbooks (17). Mechanisms that ensure access to recent clinical evidence that may influence treatment recommendations among medical students and graduate medical trainees must be in place.

This meta-analysis is limited to the time frame from the inception of the databases to June 2022 only; hence, studies done after this period will not be included. Similarly, only studies obtained using the pre-specified search engines are included in the final analysis; hence, studies which are unpublished are not included in the study. Furthermore, it may be possible that the breakdown of specific data points in the studies are not available in the main text or appendices which may obscure any further analysis or subgroup analysis.

## Data Availability

The original contributions presented in the study are included in the article/Supplementary Material, further inquiries can be directed to the corresponding author.
